# Halide metathesis in overdrive: mechanochemical synthesis of a heterometallic group 1 allyl complex

**DOI:** 10.3762/bjoc.15.181

**Published:** 2019-08-02

**Authors:** Ross F Koby, Nicholas R Rightmire, Nathan D Schley, Timothy P Hanusa, William W Brennessel

**Affiliations:** 1 Department of Chemistry, Vanderbilt University, PO Box 1822, Nashville, TN 37235, USA; 2 X-ray Crystallographic Facility, B51 Hutchison Hall, Department of Chemistry, University of Rochester, Rochester, NY 14627, USA

**Keywords:** caesium, entropy, intermolecular forces, mechanochemistry, metathesis, potassium

## Abstract

As a synthesis technique, halide metathesis (*n* RM + M'X*_n_* → R*_n_*M' + *n* MX) normally relies for its effectiveness on the favorable formation of a metal halide byproduct (MX), often aided by solubility equilibria in solution. Owing to the lack of significant thermodynamic driving forces, intra-alkali metal exchange is one of the most challenging metathetical exchanges to attempt, especially when conducted without solvent. Nevertheless, grinding together the bulky potassium allyl [KA']_∞_ (A' = [1,3-(SiMe_3_)_2_C_3_H_3_]^–^) and CsI produces the heterometallic complex [CsKA'_2_]_∞_ in low yield, which was crystallographically characterized as a coordination polymer that displays site disorder of the K^+^ and Cs^+^ ions. The entropic benefits of mixed Cs/K metal centers, but more importantly, the generation of multiple intermolecular K^…^CH_3_ and Cs^…^CH_3_ interactions in [CsKA'_2_]_∞_, enable an otherwise unfavorable halide metathesis to proceed with mechanochemical assistance. From this result, we demonstrate that ball milling and unexpected solid-state effects can permit seemingly unfavored reactions to occur.

## Introduction

Halide (or ‘salt’) metathesis is a broadly useful synthetic technique in organometallic chemistry, applicable to elements across the entire periodic table. A typical instance involves the reaction of a metal halide (M'X*_n_*) with an organoalkali metal compound (RM; M = Li, Na, K) (Equation 1) [1].

[1]
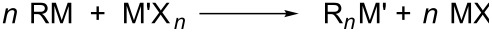


As the generation of MX normally provides a substantial portion of the energy for the exchange, M should be more electropositive than M', in order to maximize hard–soft acid–base interactions [2]. The reaction will proceed without solvent, and mechanochemical activation, which promotes reactions through grinding or milling with no, or minimal, use of solvents, has been used in conjunction with halide metathesis to form organometallic compounds of the transition metals [3–7] and both s- [8–9] and p-block [10–11] main group elements.

The extent to which the exchange represented in Equation 1 is complete varies widely with the system. In general, the larger the value of *n*, and the correspondingly increased amount of MX that is formed, the greater the driving force. Consequently, exchange will be assisted with higher valent M'X*_n_* halides. Furthermore, although in general a solvent is not required, in solution environments the formation of products is assisted if the solubility of MX or R*_n_*M' is limited, as their precipitation helps shift the equilibrium toward the product side. If ethers are used as solvents, for example, the low solubility of MX can be reduced further by choosing M to be potassium rather than lithium; as an added benefit, the resulting potassium halides are less likely to contaminate the desired product.

Without a solvent present and if M and M' are both univalent metals with similar electronegativity, complete exchange becomes difficult, and the extent of even partial exchange is hard to predict. For the alkali metals, all electronegativity scales indicate that caesium is the most electropositive, but they also indicate that there is comparatively little variation in this metric [12]. What happens when the energy difference between M'X and MX becomes particularly small? Here we describe the application of mechanochemistry in an organometallic context to examine alkali metal halide exchange unassisted by solvents. The organic group used is the bulky 1,3-bis(trimethylsilyl)allyl anion, [1,3-(SiMe_3_)_2_C_3_H_3_]^−^ ([A']^−^) [13–14], for which alkali metal complexes are known, including those of Li [15], Na [16], K [17–18], and Cs [18]. These have been formed via traditional solvent-based routes, by deprotonation of the substituted propene precursor with a metal alkyl or hydride (Equation 2) or with the metal itself (Equation 3) [18]. Intra-alkali metal exchange (although not specifically halide metathesis) has been conducted with the [A']^–^ anion, but always in the presence of a solvent to help drive the process (Equation 4) [19].

[2]
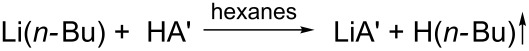


[3]
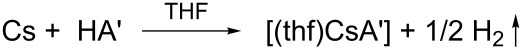


[4]
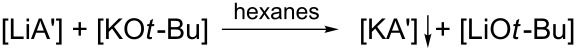


## Results and Discussion

### Conditions for halide exchange

Apart from thermodynamic considerations, practical concerns place limits on the combinations of halides and alkali metals that could be feasibly studied in intra-alkali exchange experiments. For example, the fluorides have the largest heats of formation of the alkali halides, regardless of metal, but their high lattice energies make them typically unreactive, even under mechanochemical conditions [20]. The iodides, in contrast, have the smallest lattice energies and thus should be the most easily disrupted and liable to exchange. Although several metal compounds of the allyl anion [A']^–^ were potential candidates for the present study, the need for a base-free, unsolvated complex that preferably had been crystallographically characterized limited the choice to the potassium complex [KA']_∞_. In that form [17], as well as when crystallized from THF [18], DME [21], or as described below, arenes, [KA']_∞_ retains the structure of an undulating or helical coordination polymer. Within these experimental parameters, the general reaction in Equation 5 was examined. When *n* = 1, a reaction carried to completion would result in full metal exchange, with partial exchange the outcome for any larger values of *n*.

[5]
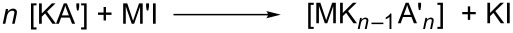


The experimental protocol involved grinding various ratios of [KA'] and alkali metal iodides, extracting the ground mixtures with hydrocarbon solvents, and then attempting crystallization of the extracts. This is necessarily an imperfect route to sampling the product space, as definitive characterization of any product(s) depended critically on the crystallizing process. In particular, NMR spectra were not expected to be highly diagnostic, as in all its group 1 complexes the resonances from the [A']^–^ anion provide a characteristic spectrum of similar chemical shifts with singlet (-SiMe_3_), doublet (C_1,3_-H), and triplet (C_2_-H) patterns that result from a π-bound allyl with *syn*,*syn*-trimethylsilyl arrangements [21].

Grinding [KA'] in a mixer or planetary mill in a 1:1 or 2:1 ratio with LiI, NaI, or RbI left the solids visibly unchanged. Only unreacted [KA'] could be extracted with toluene from the ground mixtures, and the allyl could be crystallized as its toluene solvate (see below). As a check on the consequences of halide identity, a 1:1 grind of [KA'] with LiCl was also investigated, but there was no evidence of reaction.

The grinds with CsI behaved differently from the others. A 1:1 grind for 5 min in a planetary mill left a pale orange solid that could be extracted with hexanes. When filtered and dried, the orange-brown residue displayed resonances in its ^1^H NMR spectrum corresponding to a single type of π-bonded allyl ligand, all shifted slightly (by 0.09-0.4 ppm) from those for [KA'] [21]. The material could not be purified, and repeating the grind for 10 min did not help. After grinding a 3:1 mixture of [KA']:CsI for 15 min, however, a pale yellow-orange solid was generated that could be extracted with hexanes. After being filtered, the yellow filtrate was evaporated to yield a yellow solid in low yield. Recrystallization from hexanes produced crystals that were yellow-orange; they were highly soluble in C_6_D_6_, giving a bright red solution. Single crystal X-ray analysis identified the crystals as the heterometallic complex [KCsA'_2_] (see below). The ^1^H NMR spectrum of the products from the 1:1 and 3:1 grinds were identical. It should be noted that both [KA'] and CsI are insoluble in hexanes, and the grinding clearly initiated a reaction that occurred before the first hexanes extraction.

### Structure of [CsKA'_2_]

**Figure 1:**
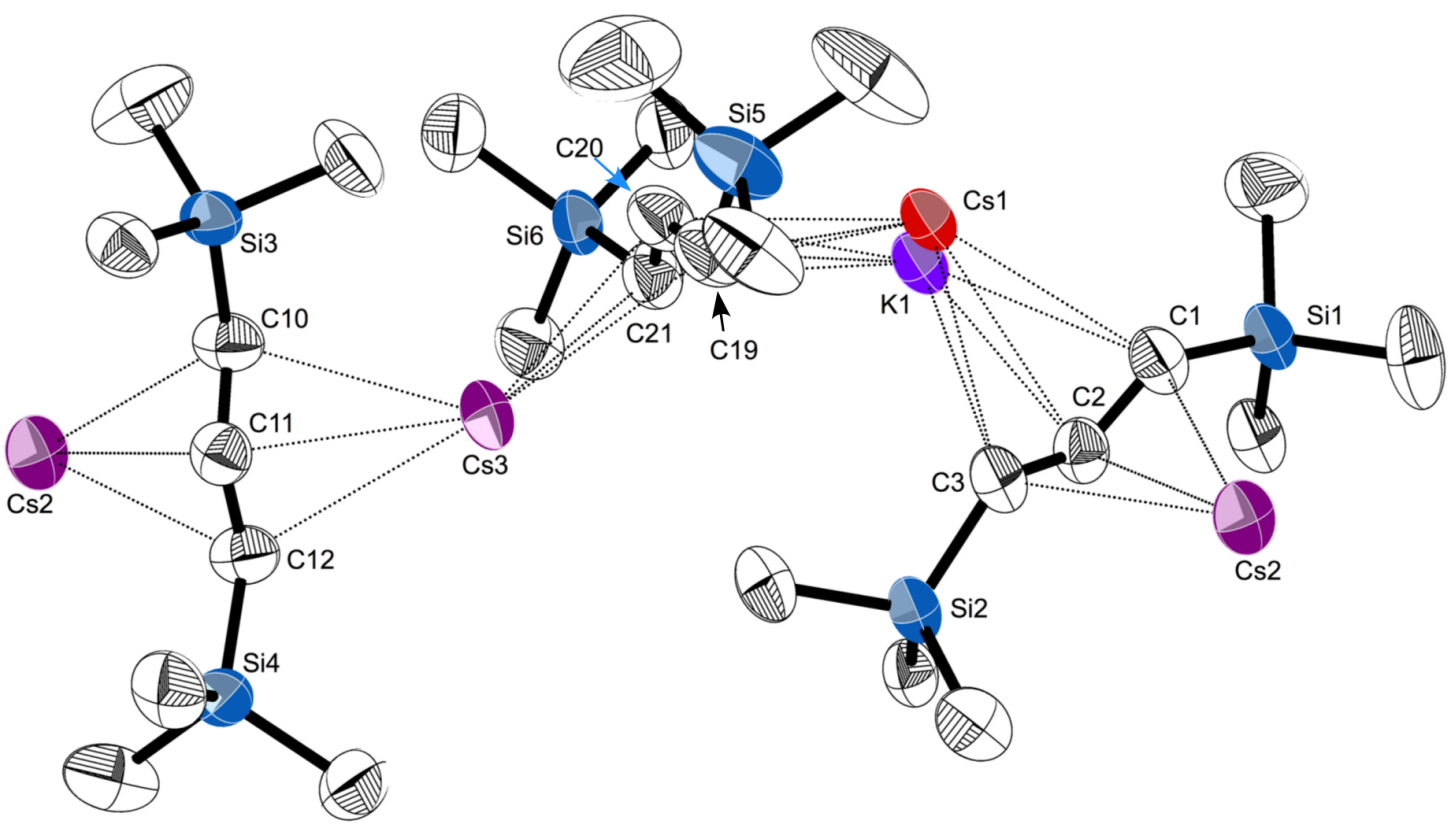
Portion of the polymeric chain of [CsKA'_2_], with thermal ellipsoids drawn at the 50% level. Hydrogen atoms have been removed for clarity. Atoms marked “Cs2” and “Cs3” are site disorders of Cs and K, with relative occupancies of 0.29:0.71 and 0.61:0.39, respectively. Selected distances (Å) and angles (deg): Cs2–C1, 3.099(4); Cs2–C2, 3.058(4); Cs2–C3, 3.198(4); Cs2–C10, 3.072(4); Cs2–C11, 2.990(4); Cs2–C12, 3.066(4); Cs1–C1, 3.242(4); Cs1–C2, 3.329(4); Cs1–C3, 3.562(4); K1–C1, 3.135(5); K1–C2, 3.026(5); Cs1–C19, 3.149(5); Cs1–C20, 3.197(4); Cs1–C21, 3.364(4); K1–C19, 3.127(6); K1–C20, 3.172(6); K1–C21, 3.184(5); Cs3–C19, 3.282(4); Cs3–C20, 3.171(4); Cs3–C21, 3.197(4); Cs3–C10, 3.184(4); Cs3–C11, 3.158(4); Cs3–C12, 3.349(4); C1–C2–C3, 130.5(4); C19–C20–C21, 129.7(4); C10–C11–C12, 131.2(4).

**Figure 2:**
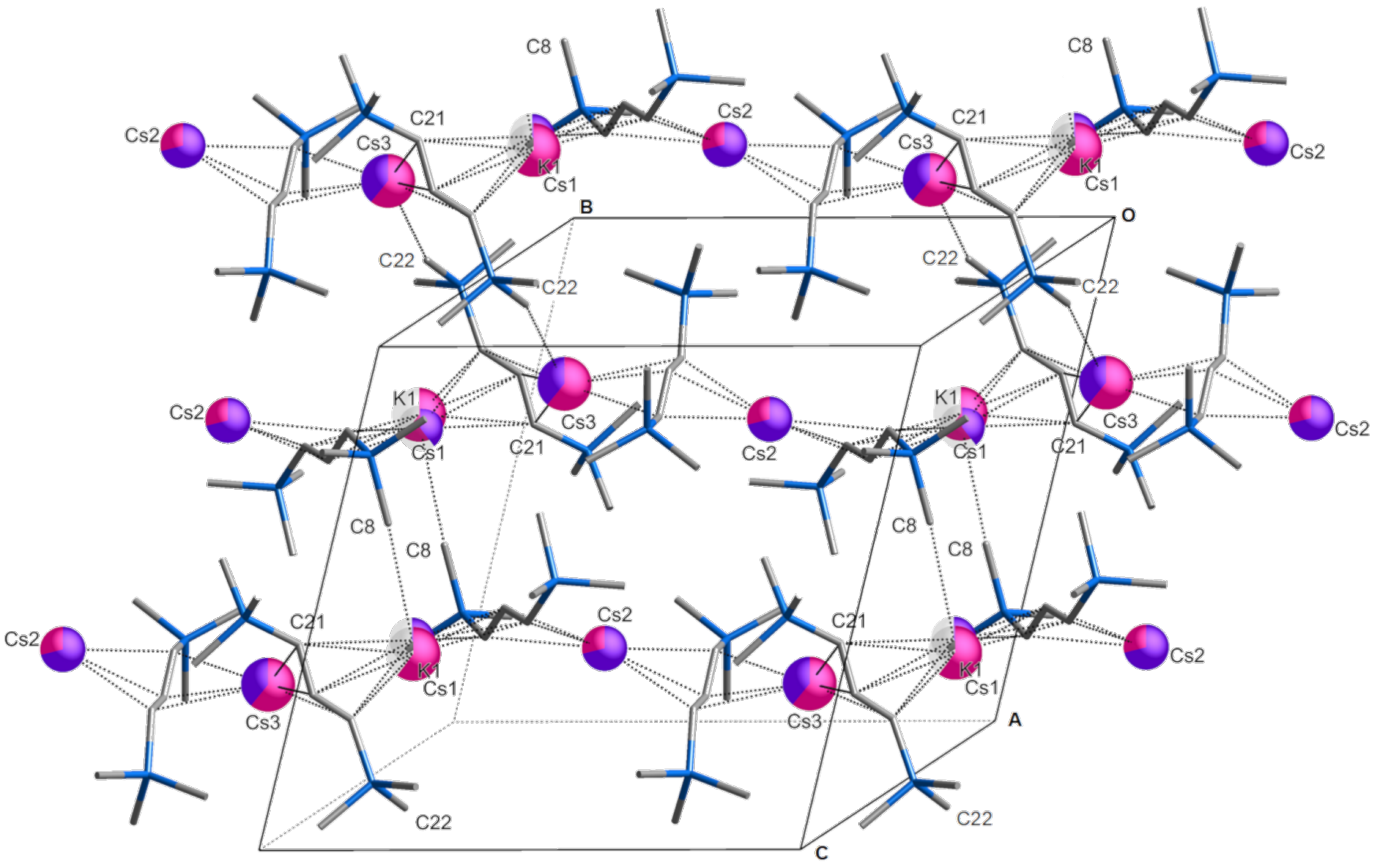
Partial packing diagram of [CsKA'_2_], illustrating some of the interchain contacts, predominantly K1^…^C8 at 3.20 Å, and Cs3^…^C22 at 3.44 Å, that promote sheet formation. The metal centers are colored in a pie chart fashion according to the proportion of K^+^ (purple) or Cs^+^ (pink) of each; translucent wedges (visible on K1 and Cs1) indicate the percentage of partial vacancy at the site. The C–C and C–Si bonds are rendered as sticks.

Small blocks grown from hexanes were identified from a single crystal X-ray study as the coordination polymer [CsKA'_2_]_∞_. A depiction of a single chain is provided in Figure 1, and a partial packing diagram is given in Figure 2. The asymmetric unit contains three alkali metal cations and three substituted allyl anions, all in general positions. Each of the three metal sites is modeled as a site disorder of atoms types K and Cs. Two distinct peaks were found in the difference Fourier map for the site containing atoms Cs1 and K1, and their positions were refined freely, but their anisotropic displacement parameters were constrained to be equivalent. For the other two site disorders (atom pairs Cs2/K2 and Cs3/K3), the atoms were constrained to be isopositional and their anisotropic displacement parameters were constrained to be equivalent. The ratios of Cs to K in the three sites refined to 0.60:0.40, 0.29:0.71, and 0.61:0.39 for atom pairs Cs1/K1, Cs2/K2, and Cs3/K3, respectively.

Although the metal–C(allyl) distances span a large range, they do so in a way that reflects the proportion of Cs and K in the metal to which they are bonded. For example, the average distance of Cs2 (0.29 Cs:0.71 K) to the allyl carbons C10–C12 is 3.04 Å. The same allyl is also bonded to Cs3, with a higher percentage of Cs (0.61 Cs:0.39 K), and the average M–C distance is correspondingly longer, at 3.22 Å. It is possible to extract consistent values from the M–C distances that can be assigned to the proportion of K and Cs, namely 2.95 Å and 3.40 Å, respectively (i.e., a hypothetical site that is 0.50 (K):0.50 (Cs) would be expected to exhibit an average M–C(allyl) bond distance of roughly 3.17 Å). These values do not recreate distances in the homometallic complexes exactly (i.e., the average K–C distance in [KA']_∞_ is 3.01 Å) [17], but they reflect the relative sizes of the K^+^ and Cs^+^ cations.

The structure is polymeric in two dimensions in the crystallographic *bc* plane; interchain K^…^CH_3_ and Cs^…^CH_3_ contacts are responsible for generating the 2D arrangement (Figure 2); this is discussed in more detail below.

### Structure of [(C_6_H_6_)KA']_∞_

From all the grinds of [KA'] with the alkali metal iodides (excepting CsI), the potassium allyl was the only recoverable material; extracted with toluene, it crystallized from solution as the solvate. A single crystal X-ray study analysis reveals bent polymeric chains of alternating K^+^ cations and [A']^−^ anions. Each potassium is capped with a toluene molecule, bonded through cation–π interactions. The structure suffers from twinning, disorder in the toluene, and weak diffraction, and therefore its structural details are degraded (a depiction of the coordination polymer is available in Supporting Information File 1). Fortunately, when [KA'] is dissolved in benzene and the solution evaporated, an analogous solvate is obtained, and the resulting crystals are of higher quality than those from toluene. Single crystal X-ray analysis reveals that it has a structure that is qualitatively the same as the toluene solvate, and only the benzene solvate is discussed here.

**Figure 3:**
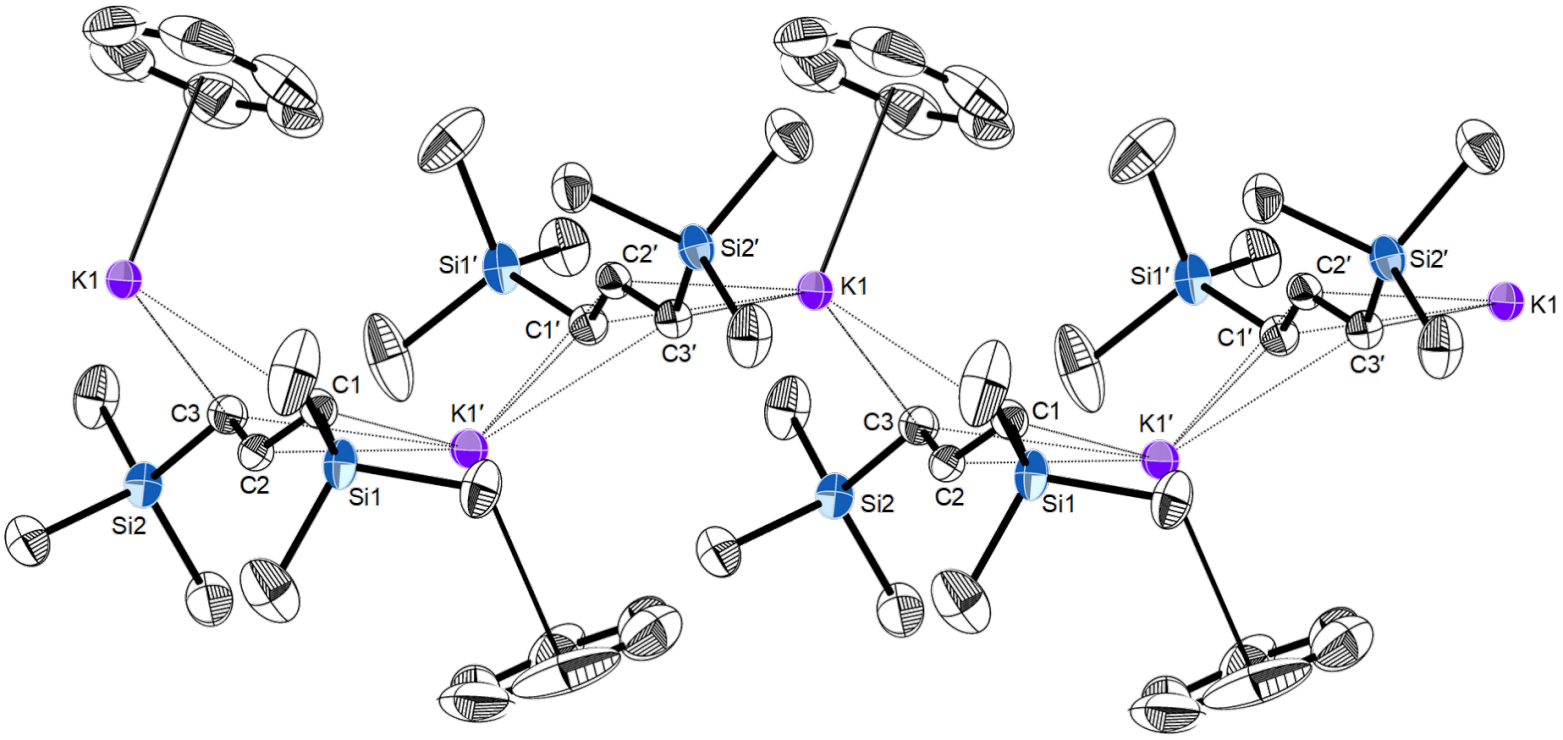
Portion of the polymeric chain of [(C_6_H_6_)KA']_∞_, with thermal ellipsoids drawn at the 50% level. Hydrogen atoms have been removed for clarity. Selected distances (Å) and angles (deg): K1–C1, 3.005(3); K1–C2, 2.963(3); K3–C3, 3.128(3); K1–C1', 2.959(3); K1–C2', 2.983(2); K3–C3', 3.140(3); K1^…^(C_6_H_6_ centroid), 2.99(1); K1^…^K1', 5.39; C1–C2–C3, 130.8(3); K1^…^K1'^…^K1, 134.0.

Like the unsolvated complex [KA']_∞_ [17] and the related DME and THF solvates [K(dme)A']_∞_ [21] and [K(thf)_3/2_A']_∞_ [18], respectively, [(C_6_H_6_)KA']_∞_ is a coordination polymer with potassium ions linked by bridging π-allyl ligands. The polymer takes the form of bent chains running parallel to the *b* axis (Figure 3). There is only one crystallographically distinct potassium ion in the chains, and a single K^…^K'^…^K bending angle of 134.0°. This is different from the pattern found in [K(thf)_3/2_A']_∞_, for example, (i.e., roughly linear K(1)^…^K(2)^…^K(1)' sections (170.2°) alternating with strongly bent K(2)^…^K(1)^…^K(2)' angles (103.3°)). The K–C_6_H_6_ ring centroid distance is 2.99 Å, which is typical for K^+…^(arene) cation–π interactions [22–23]. The enthalpy of binding (∆*H°*) of an isolated K^…^(benzene or toluene) unit is almost 80 kJ mol^−1^ (see calculated value in Table 1, entry 5); the energy is reduced by about 40% when the ring is bound to the neutral [KA'] fragment (entry 6).

**Table 1:** Energies of reaction (B3PW91-D3BJ, kJ mol^−1^).

Entry	Reaction^a^	Energy (∆*H*°, ∆*G*°)

1	K^+^ + [C_3_H_5_]^−^ → [K(C_3_H_5_)]	−514.6, −481.5
2	Cs^+^ + [C_3_H_5_]^−^ → [Cs(C_3_H_5_)]	−484.9, −452.2
3	K^+^ + [A']^−^ → [KA']	−458.4, −426.5
4	Cs^+^ + [A']^−^ → [CsA']	−430.4, −398.1
5	K^+^ + toluene → [(toluene)K]^+^	−78.9, −48.6
6	[KA'] + toluene → [(toluene)KA']	−48.1, −13.1

^a^For entries 1–4, the def2-TZVPD basis set was used on all atoms. For entries 5 and 6, the def2-TZVP basis set was used on all atoms.

In structurally characterized polymeric [L*_n_*KA']_∞_ complexes, the average K–C(allyl) distances span a comparatively narrow range, regardless of coordinated ligands and the change in formal coordination number of the K^+^ cation: i.e., 3.01 Å in [KA']_∞_, 3.03 Å in [KA'(thf)_3/2_]_∞_ and [(C_6_H_6_)KA']_∞_ (3.04 Å in the poorer quality [(toluene)KA']_∞_ structure), and 3.06 Å in [KA'(dme)]_∞_ [24]. This suggests that the K^+…^[A'] interaction is a robust one, and its structure potentially capable of serving as a kind of template for inclusion (see below).

### Formation of the heterometallic allyl complex

The net reaction that produced the clearest evidence for mechanochemically promoted alkali metal exchange is given by Equation 5 with *n* = 2 (i.e., Equation 6). Several features of it are noteworthy.

[6]
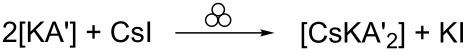


The ratio of [KA'] to CsI that yielded [CsKA'_2_] was produced both from a 1:1 and a 3:1 ratio of [KA'] to CsI, and the predicted result from Equation 5, assuming complete reaction, would have been either pure [CsA'] or the heterometallic [CsK_2_A'_3_]. That neither of these outcomes was observed, and a non-stoichiometric product was obtained is actually not uncommon in mechanochemical synthesis, and can reflect the fact that products often do not have time to equilibrate or go from metastable to more stable products [9,11]. There can be multiple reasons for this, starting with the high energy environment of grinding that may be far from equilibrium [25], allowing the kinetic products to be the ones most likely to be isolated. The high concentration of reagents in a solid-state reaction may influence product formation as well. The possibility of partial exchange also needs to be considered. If the caesium iodide were insufficiently reactive, a starting ratio of 3:1 for [KA']:CsI could give rise to products with higher ratios of K to Cs than even [CsK_2_A'_3_], such as [CsK_3_A'_4_] or [CsK_4_A'_5_]. In this light, it is notable that CsI is the limiting reagent in the reaction, and the resulting 1:1 ratio of the metals in the allyl complex suggests that it is a favored composition.

Secondly, the relative free energies of formation of CsI and KI (−341 and −325 kJ mol^−1^, respectively; ∆∆*G* = +16 kJ mol^−1^) [26] means that the formation of the metal halide byproduct (KI) is non-spontaneous, and does not contribute to the driving force for the reaction. The relative free energies of the allyl complexes then must provide the difference. There are no experimental values available for the thermodynamic quantities involving potassium and caesium allyls, however, although it would be expected that the smaller K^+^ ion would interact more strongly with the allyl anion than would the larger, softer Cs^+^ ion.

To explore this and several related points more quantitatively, various features of the K/Cs/[allyl]^−^ system were modeled with DFT calculations, using the B3PW91 hybrid functional [27–28] with Grimme’s -D3 dispersion corrections (GD3BJ) [29]. A calculation on the simple model systems [K(C_3_H_5_)] and [Cs(C_3_H_5_)] indicates that, consistent with the above rationale, ∆*G*°_f_ for the potassium complex is more negative than for the caesium complex (by 29.3 kJ mol^−1^; Table 1, entries 1 and 2). The slightly greater realism provided by comparing the [KA'] and [CsA'] complexes does not meaningfully affect the difference (28.4 kJ mol^−1^; Table 1, entries 3 and 4). Of course, these are calculations on isolated monomers, and the energetics of formation of the solid-state polymeric forms [30] would be expected to change these values, but not necessarily in a way that would clearly favor the formation of [CsA'] over [KA']. If so, there would consequently be no thermodynamic driving force for the metathesis reaction.

There are several ways that this simple analysis underestimates the energetics involved in the system. For example, full metal exchange does not occur, and the resulting heterometallic allyl complex has additional entropy provided by the two metal ions and the site disorder in the solid. Using a standard formula for the entropy of mixing two species (configurational entropy, ∆*S*_mix_ = −*nR*(*X*_A_ ln *X*_A_ + *X*_B_ ln *X*_B_) [31], and with 3 atoms distributed randomly over the three crystallographically identified metal sites, the value of ∆*S* = +17 J mol^−1^ K^−1^ is obtained. At 298 K, the −*T*∆*S* value is −5.1 kJ mol^−1^. As imperfect as this approximation is (e.g., the distribution of metal ions is not completely random, and the coordination environments are not exactly the same), it does suggest one source of driving force not present in the homometallic allyls.

A potentially much more important source of stability in [CsKA'_2_] is the existence of multiple intermolecular M^…^CH_3_ interactions, including Cs^…^CH_3_ contacts, obviously energetically significant enough that they support the formation of two-dimensional sheets in the solid state. To appreciate the magnitude of this effect, the relative conformation of the known [L*_n_*KA'] complexes are summarized by their (non-bonded) K^…^K'^…^K angles (Table 2).

**Table 2:** Non-bonded intrachain K^…^K'^…^K angles in [L*_n_*KA'] complexes.

Complex	K^…^K'^…^K (deg)	Reference

[KA']_∞_	135.1; 135.7; 118.2	[17]
[K(dme)A']_∞_	153.3, 141.9	[21]
[K(dme)A']_∞_	170.0, 103.3	[18]
[(C_6_H_6_)KA']_∞_	134.0	this work
[KCsA'_2_]_∞_	140.3 (K1–Cs2–Cs3); 141.0 (K1–Cs3–Cs2); 107.3 (Cs2–K1–Cs3)	this work

Although the K^…^K'^…^K angles are only markers (there are no direct K^…^K' interactions in any of the complexes), it is notable that both [KA']_∞_ and [CsKA'_2_] display three such angles, two of which are relatively similar at ca. 135–140°, and a third that is substantially more bent (<120°) (see the Supporting Information File 1, Figure S2, for a visualization of the similarity). The significance of this is that [KA'] can be viewed as a template into which Cs^+^ are infused during the grinding. There are adjustments in M–C(allyl) bond distances (see above), but another consequence is the generation of multiple intermolecular M^…^CH_3_ interactions. Both [KA'] and [CsKA'_2_] possess K^…^CH_3_ contacts at typical distances [32]; in [KA'], the two closest are both at 3.23 Å; the third is at 3.35 Å. In [CsKA'_2_], the closest is at 3.20 Å, with the second at 3.38 Å.

The Cs^…^CH_3_ interactions in [CsKA'_2_], however, are especially noteworthy. The closest is at 3.44 Å (Cs3^…^C22), followed by four more at 3.56 Å, and farther ones at 3.67 and 3.74 Å. All these distances are substantially shorter than the sum of the van der Waal’s radii of Cs (3.43 Å) and CH_3_ (2.00 Å). Intermolecular Cs^…^CH_3_ distances of ca. 3.6 Å and longer are not especially rare, and are strong enough to influence solid state structures. In the dme adduct of caesium [2,4,6-tri(*tert*-butyl)phenolate], for example, a Cs^…^CH_3_ contact of 3.596(5) Å contributes to its form as a 1D coordination polymer [33]. In the caesium salt of the gallium metallate [Cs(toluene)_2_{CN(GaMe_3_)_2_}], multiple Cs^…^CH_3_ interactions in the range from 3.54–3.64 Å help generate its three-dimensional network structure [34]. Intermolecular Cs^…^CH_3_ distances below 3.5 Å, however, do not appear to have been previously reported [35]. The shortest distance in [CsKA'_2_], at 3.44 Å, is 2.0 Å less than the sum of the appropriate van der Waal’s radii (although less precisely located, the corresponding Cs^…^H distance (Cs3^…^H22B) is 3.05 Å, a third less than the sum of the van der Waal’s radii (4.63 Å).

Calculations on the model systems [(CH_4_)(K,Cs)A'] and [(HMe_2_SiMe)(K,Cs)A'] were used to place the energy of the M^…^methyl interactions in context (views of the optimized pairs are available in the Supporting Information File 1, Figure S3). Despite the gas-phase environment of the calculations, the distance between K^+^ and CH_4_ is 3.22 Å, a typical value for potassium–methyl interactions in the solid state, as is the ∆*H*° of almost 12 kJ mol^−1^, in the range of hydrogen bonds (Table 3, entries 1 and 2) [32]. The distance of K^+^ to Me_3_SiH, chosen to represent somewhat more accurately the type of interactions occurring in [KCsA'_2_], is slightly shorter (3.14 Å) and stronger (30 kJ mol^–1^), probably a result of the lower electronegativity of silicon compared to carbon and the correspondingly more negative methyl groups. The analogous calculations with Cs^+^ (Table 3, entries 3 and 4) place the contact distance at 3.62 Å and 3.53 Å for CH_4_ and Me_3_SiH, respectively, with corresponding enthalpies of −3.9 and −23.6 kJ mol^−1^. These distances are similar to those found in the solid state, and together with the potassium interactions, evidently help to drive the heterometallic complex formation.

**Table 3:** Energies of reaction (B3PW91-D3BJ, kJ mol^−1^

Entry	Reaction^a^	Energy

1	[KA'] + [CH_4_] → [(CH_4_)']	−11.9 (∆*H*°)
2	[KA'] + HSiMe_3_ → [(HSiMe_3_)KA']	−30.0 (∆*H*°)
3	[CsA'] + CH_4_ → [(CH_4_)CsA']	−3.9 (∆*H*°)
4	[CsA'] + HSiMe_3_ → [(HSiMe_3_)CsA']	−23.6 (∆*H*°)

^a^The def2-TZVP basis set was used on all atoms.

## Conclusion

Formally, halide metathesis as a synthetic technique depends strongly on the relative thermodynamic stabilities of the starting and final metal halide salts, M'X and MX. Practically, however, the reaction solvent is also a critical assistant in the process, as the insolubility of the MX product can strongly shift the position of equilibrium and drive the reaction. Mechanochemical techniques can be used to provide a driving force for a reaction that would be energetically unfavorable and has no solvent assistance. The formation of the heterometallic [CsKA'_2_] from the mixture of [KA'] and CsI, even though in low yield, owes its realization to the entropic benefit of a mixed metal system, but even more importantly to the formation of intermolecular M^…^CH_3_ contacts, permitting the formation and stabilization of a sheet structure that ties the coordination polymer chains of M^…^A' units together. Recognition of this additional source of reaction energy has the potential to extend the usefulness of halide metathesis to systems previously considered too unpromising to explore.

## Supporting Information

Crystallographic data for the structures reported in this paper have been deposited with the Cambridge Crystallographic Data Centre as CCDC 1897690 ([KCsA'_2_]), 1897691 ([(C_6_H_6_)KA']), and 1897692 ([(toluene)KA']). Copies of the data can be obtained free of charge on application to CCDC, 12 Union Road, Cambridge CB2 1EZ, UK (fax: (+44)1223-336-033; email: deposit@ccdc.cam.ac.uk).

File 1 Experimental and computational details; crystal data and summary of X–ray data collection.

File 2 Coordinates of DFT-optimized structures.
