# Supplementary material for: Halide metathesis in overdrive: mechanochemical synthesis of a heterometallic group 1 allyl complex
Source: Beilstein J Org Chem. 2019 Aug 2;15:1856–63. doi: 10.3762/bjoc.15.181 (PMC6693392; doi:10.3762/bjoc.15.181)
Supplement: File 1 — Experimental and computational details; crystal data and summary of X–ray data collection. [file Beilstein_J_Org_Chem-15-1856-s001.pdf]

BEILSTEIN JOURNAL OF ORGANIC CHEMISTRY

Supporting Information

for

Halide metathesis in overdrive: mechanochemical synthesis of a heterometallic group 1 allyl complex

Ross F. Koby, Nicholas R. Rightmire, Nathan D. Schley, Timothy P. Hanusa and William W. Brennessel

Beilstein J. Org. Chem. **2019,** *15,* 1856–1863. doi:10.3762/bjoc.15.181

Experimental and computational details; crystal data and summary of X–ray data collection

License and Terms: This is a supporting information file under the terms of the Creative Commons Attribution License (http://creativecommons.org/licenses/by/4.0). Please note that the reuse, redistribution and reproduction in particular requires that the authors and source are credited.
The license is subject to the *Beilstein Journal of Organic Chemistry* terms and conditions: (https://www.beilstein-journals.org/bjoc)

Table of contents

Synthetic procedures S2

Procedures for X-ray crystallography S4

General procedures for calculations S4

Figure S1: Connectivity of the coordination polymer chain in [(toluene)KA´]$_\infty$ S5

Figure S2: Superposition of a portion of the structures of [KA´]$_\infty$ and [KCsA´$_2$]$_\infty$ S6

Figure S3: Calculated geometries of [MA´]···Me interactions S7

Table S1: Crystal data and summary of X–ray data collection S8

References S9

Synthetic procedures

General considerations

All syntheses were conducted under rigorous exclusion of air and moisture using Schlenk line and glovebox techniques. Proton (^{1}H) and carbon (^{13}C) spectra were obtained on an Advance AV-400 MHz spectrometer at 400 and 100 MHz, respectively. Proton and carbon were referenced to residual resonances of C_6D_6.

Materials

All anhydrous metal salts and were purchased from Strem or Sigma-Aldrich, stored under an N_2 atmosphere, and used as received. The potassium allyl [KA′] = [K(1,3-$SiMe_3$)C_3H_3] was synthesized by transmetalation of [LiA′] [1] with potassium *tert*-butoxide in hexanes solution. Toluene was degassed with argon and dried over activated alumina using a solvent purification system, then stored over 4 Å molecular sieves in a glovebox. Hexanes were distilled under nitrogen over NaK/benzophenone radical [2[, then stored over 4 Å molecular sieves in a glovebox. Benzene-d_6 was obtained from Cambridge Isotopes and stored over 4 Å molecular sieves.

Mechanochemical protocol. Planetary milling was performed with a Retsch PM100 mill, 50 mL stainless steel grinding jar type C, and a safety clamp for air-sensitive grinding. Mixer milling was performed with a Retsch model MM200 mill. Ball milling reactions used 50 stainless steel (440 grade) ball bearings ($^{3}/_{16}$ in (5 mm), 0.44 g) or 3 stainless steel (440 grade) ball bearings ($^{1}/_{2}$ in (12.7 mm), 8.4 g) that were thoroughly cleaned with detergent and water, then washed with acetone, and dried in a 125 °C oven prior to use. A typical reaction was sealed under an inert atmosphere prior to grinding. The ground mixture was extracted with minimal hexanes (<100 mL) and filtered through a medium porosity ground glass frit. The extraction is designed to dissolve the complex, and the filtration removes residual KI. The filtrate was then dried under vacuum prior to NMR analysis.

Synthesis of [CsKA′$_2$]. Solid CsI (0.157 g, 0.604 mmol) and K[A′] (0.445 g, 1.98 mmol) were added to a 50 mL stainless steel grinding jar (type C). The jar was charged with stainless steel ball bearings (½ in dia, 3 count) and closed tightly with the appropriate safety closer device under an N_2 atmosphere. The reagents were milled for 15 min at 600 rpm, resulting in a pale yellow-orange solid. The solid was extracted with hexanes and filtered through a medium porosity ground glass frit, providing a yellow-tinted filtrate. Removal of hexanes under vacuum resulted in a turbid

yellow solution that deposited a precipitate as the product was concentrated. Removal of hexanes under vacuum yielded a yellow solid in low yield (12 mg, 4% yield). The solid was dissolved in hexanes, and the solution slowly evaporated over the course of a week to promote crystal growth. As the concentration of the solution increased, it became more orange. X-ray analysis of the crystals revealed them to be the bimetallic [CsKA′$_2$] complex. The crystals were highly soluble in C_6D_6, giving a bright red solution. ^{1}H NMR (400 MHz, C_6D_6, 298 K): δ 0.27 (s, 18H, $SiMe_3$); 2.71 (d, 2H, J = 16.0 Hz, $H_{(\alpha,\gamma)}$); 6.58 (t, 1H, J = 16.0 Hz, $H_{(\beta)}$). ^{13}C NMR (100 MHz, C_6D_6, 298K): δ 2.32 ($SiMe_3$); 73.08 ($C_{(\alpha,\gamma)}$); 153.51 ($C_{(\beta)}$).

Procedures for X-ray crystallography

[$K_{1.5}Cs_{1.5}(1,3\text{-}(SiMe_3)_2C_3H_3)_3$]

A crystal was placed onto the tip of a thin glass optical fiber and mounted on a Bruker SMART APEX II CCD platform diffractometer at the X-ray Crystallographic Facility, Department of Chemistry, University of Rochester (Rochester, NY). Data collection was conducted at 100 K using MoKα radiation (graphite monochromator) [3]. The structure was solved using SHELXT-2014/5 [4] and refined using SHELXL-2014/7 [5]. The space group $P\overline{1}$ was determined based on intensity statistics. A direct-methods solution was calculated which provided most non-hydrogen atoms from the E-map. Full-matrix least squares/difference Fourier cycles were performed which located the remaining non-hydrogen atoms. All non-hydrogen atoms were refined with anisotropic displacement parameters. Hydrogen atoms on the allylic portions of the anions were found from the difference Fourier map and their positions were refined independently from those of their respective bonded carbon atoms. However, their isotropic displacement parameters were refined relative to the (equivalent) anisotropic displacement parameters of their respective bonded carbon atoms. All other hydrogen atoms were placed in ideal positions and refined as riding atoms with relative isotropic displacement parameters.

[(Benzene)K($1,3\text{-}(SiMe_3)_2C_3H_3$)], [(toluene)K($1,3\text{-}(SiMe_3)_2C_3H_3$)]

X-ray crystallographic data were collected on a Rigaku Oxford Diffraction Supernova diffractometer. Crystal samples were handled under immersion oil and quickly transferred to a cold nitrogen stream. The crystals were kept at 100 K during data collection. Under Olex2 [6], the structure was solved with the SHELXT[7] structure solution program using direct methods and refined with the SHELXL [5] refinement package using least squares minimization. All non-hydrogen atoms were refined with anisotropic displacement parameters.

General procedures for calculations

All calculations were performed with the Gaussian 09W [8] or Gaussian 16 (Linux) suite of programs [9]. The B3PW91 functional, which incorporates Becke's three-parameter exchange functional [10] with the 1991 gradient-corrected correlation functional of Perdew and Wang [11], was used. For dispersion-corrected calculations, Grimme's D3 correction [12] with additional Becke-Johnson damping was used [13] (Gaussian keyword: empiricaldispersion=GD3BJ). For the energy of metal allyl complex formation ($M^+ + [A']^- \rightarrow [MA']$), the def2-TZVPD basis set was used on all atoms, with the accompanying ECP used for Cs [14]. For all other calculations, the def2-TZVP basis set was used on all atoms, with an ECP on Cs. An ultrafine grid was used for all calculations (Gaussian keyword: int=ultrafine).

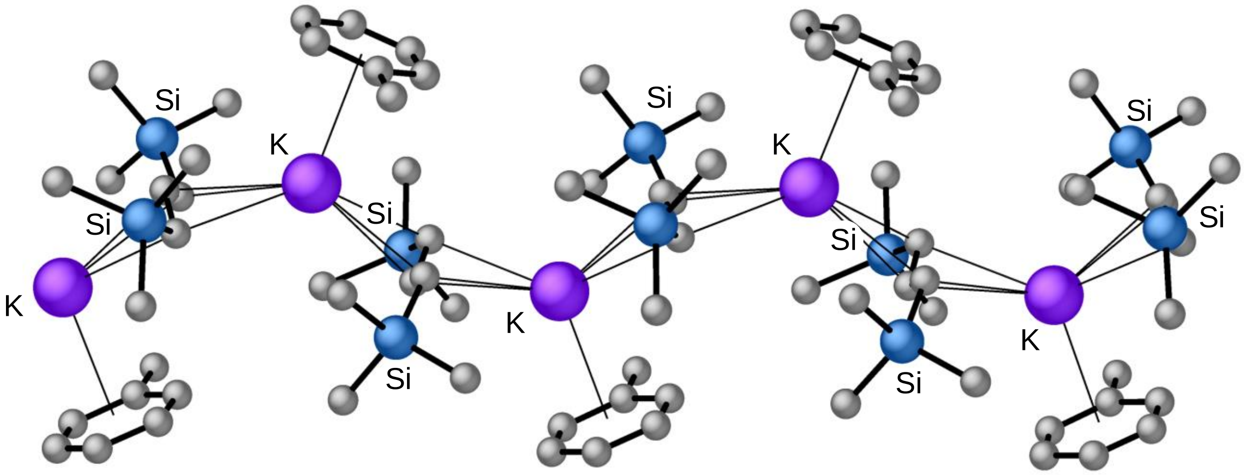

Figure S1. Connectivity of a portion of the coordination polymer chain in [(toluene)KA´]$_{\infty}$. Only one orientation of the toluene ligand is shown. Approximate bond distances (Å) and angles (deg): K···K´= 5.52; K–C(allyl) (ave) = 3.04; K–ring centroid = 3.01; K···K´···K = 130.

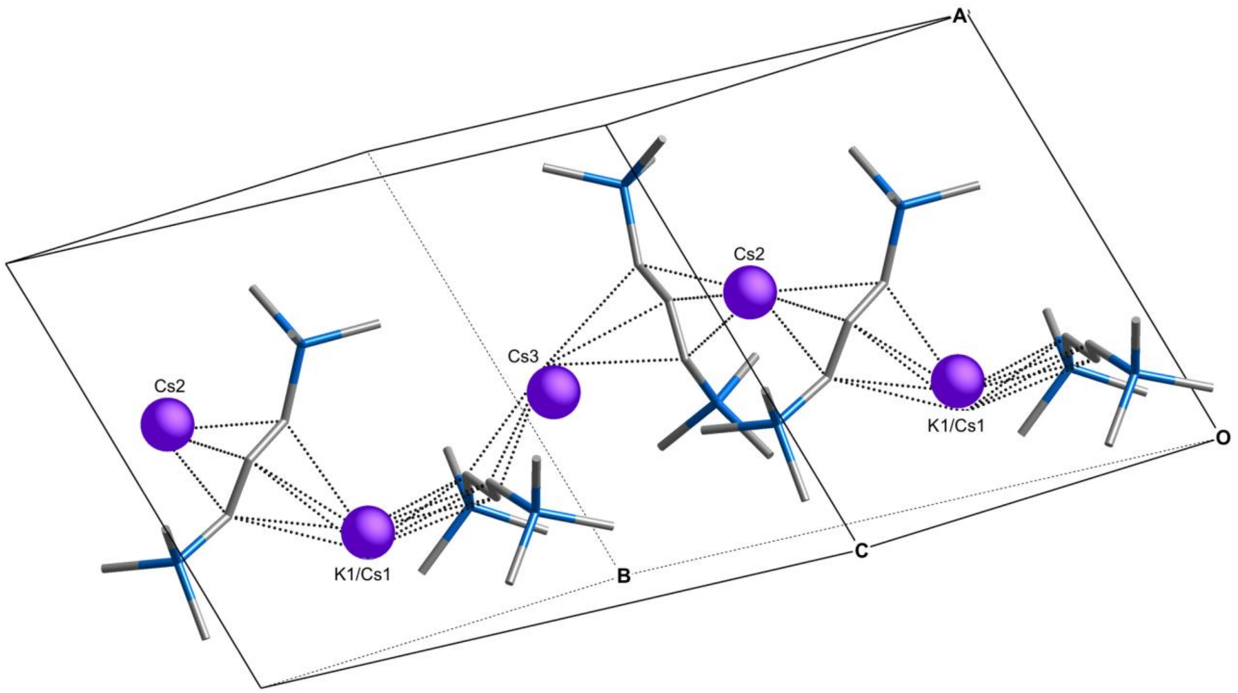

Figure S2. Superposition of a portion of the structures of [KA´]$_\infty$ and [KCsA´$_2$]$_\infty$ (the unit cell is from [KCsA´$_2$]). From [KA´]$_\infty$, only the potassium atoms are shown; from [KCsA´$_2$]$_\infty$, only the allyl ligands (as sticks) are shown, and the dotted lines are drawn to where the disordered potassium/cesium sites would be located. The overlap of the metal sites is not exact, and this view is meant to convey only a general sense of the alignment. For exact M···M´···M´´ angles, see the main article.

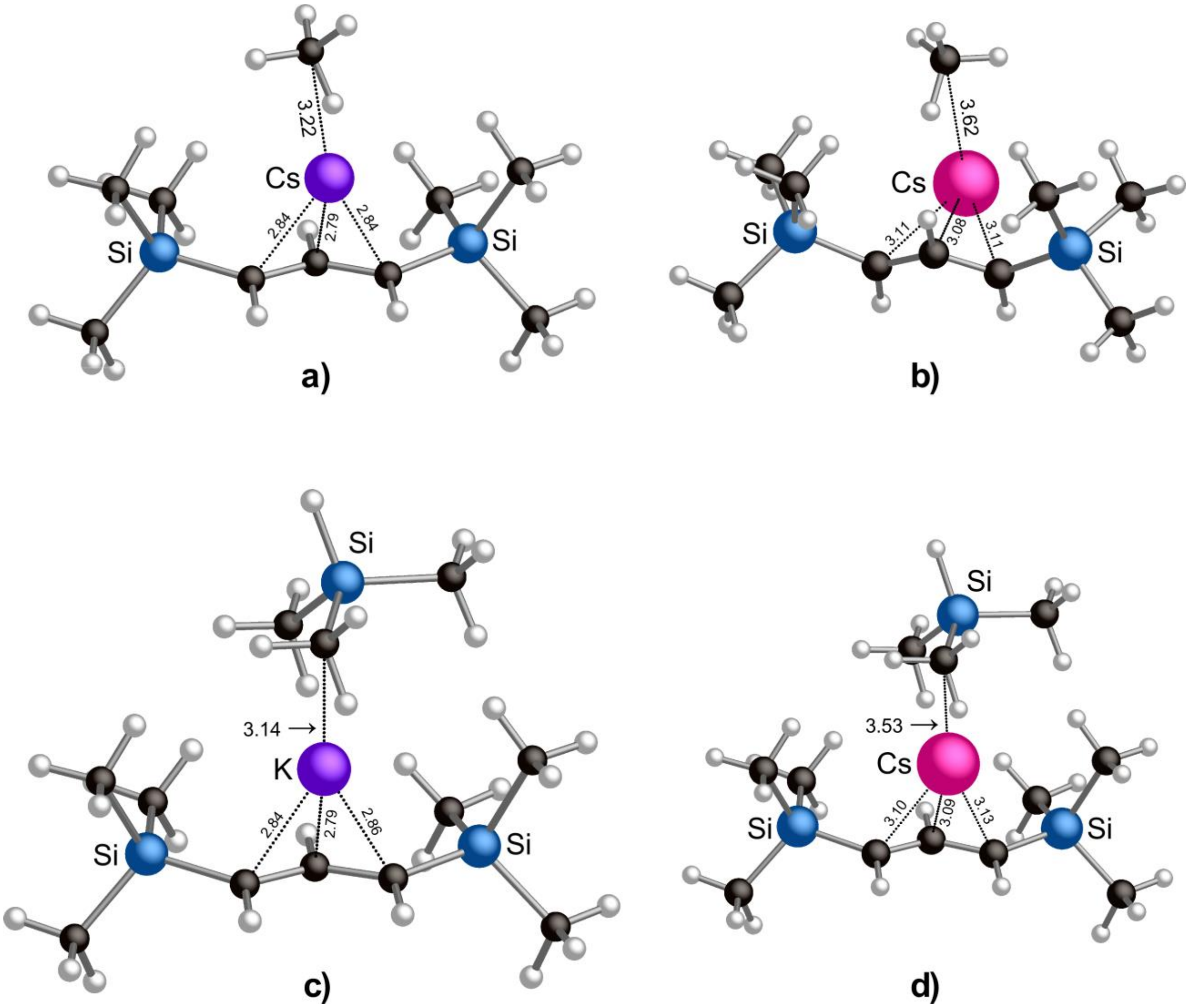

Figure S3. Calculated geometries of [MA´]···Me interactions in: a) [KA´]···CH_4; b) [CsA´]···CH_4; c) [KA´]···$MeSiMe_2H$; d) [CsA´]···$MeSiMe_2H$. For energies associated with the interactions, see the main article.

Table S1: Crystal data and summary of X–ray data collection

**Compound**	**$[K_{1.5}Cs_{1.5}(1,3\text{-}(SiMe_3)_2C_3H_3)_3]$**	**$[(benzene)K(1,3\text{-}(SiMe_3)_2C_3H_3)]$**	**$[(toluene)K(1,3\text{-}(SiMe_3)_2C_3H_3)]$**
Empirical formula	$C_{27}H_{63}Cs_{1.5}K_{1.5}Si_6$	$C_{15}H_{27}KSi_2$	$C_{16}H_{29}KSi_2$
Formula weight	814.33	302.64	316.67
Color of compound	yellow	colorless	colorless
Temperature/K	100.0(5)	100	100.01(10)
Crystal system	triclinic	monoclinic	monoclinic
Space group	$P\bar{1}$	$P2_1$	$P2_1/n$
a/Å	11.1741(8)	10.1620(3)	10.2297(11)
b/Å	14.6733(11)	9.93170(18)	10.1301(10)
c/Å	14.7968(11)	10.7700(3)	19.127(2)
α/°	73.9101(15)	90	90
β/°	85.2391(16)	118.137(3)	91.119(10)
γ/°	71.6969(15)	90	90
Volume/Å^3	2213.1(3) Å	958.52(5)	1981.7(4)
Z	2	2	4
ρ_{calc} g/cm^3	1.222	1.049	1.061
μ/mm^{-1}	1.559	3.486	3.391
F(000)	840	328	688
Crystal size/mm^3	0.25 × 0.16 × 0.14	0.316 × 0.185 × 0.073	0.1 × 0.05 × 0.03
Radiation	MoKα (λ = 0.71073)	CuKα (λ = 1.54184)	CuKα (λ = 1.54184)
2Θ range for data collect/°	1.788 to 27.500	4.452 to 73.258	4.625 to 65.078
Index ranges	-14≤ *h* ≤ 14, -19 ≤ *k* ≤ 19, -19 ≤ *l* ≤ 19	-12 ≤ *h* ≤ 12, -12 ≤ *k* ≤ 12, -12 ≤ *l* ≤ 12	-12 ≤ *h* ≤ 12, -11 ≤ *k* ≤ 11, 0 ≤ *l* ≤ 22
Reflections collected	29 788	13 244	3365
Independent reflections	10133 [R_{int} = 0.0374]	3762 [R_{int} = 0.0269]	3365
Data/restraints/parameters	10133/43/404	3762/4/181	3365/180
Goodness-of-fit on F^2	1.046	1.031	1.069
Final *R* indexes [*I* > 2σ (*I*)]	R_1 = 0.0445, wR_2 = 0.1080	R_1 = 0.0209, wR_2 = 0.0539	R_1 = 0.1055, wR_2 = 0.3272
Final *R* indexes [all data]	R1 = 0.0746, wR2 = 0.1234	R_1 = 0.0210, wR_2 = 0.0541	R1 = 0.1271, wR2 = 0.3441
Largest diff. peak/hole/e Å^{-3}	0.938/-0.622	1.42/-0.80	1.51/-0.77

References

1. Fraenkel, G.; Chow, A.; Winchester, W. R. *J. Am. Chem. Soc.*, **1990**, *112*, 1382-1386.
2. Armerego, W. L. F.; D. D. Perrin, D. D. *Purification of Laboratory Chemicals*, 4th ed.; Pergamon: Oxford, 1997; p 16
3. *APEX3*, version 2015.5-2; Bruker AXS: Madison, WI, 2015.
4. Sheldrick, G. M. *SHELXT-2014/5*; University of Göttingen: Göttingen, Germany, 2014.
5. Sheldrick, G. M. *SHELXL-2014/7*; *Acta Crystallogr. Sect. C*, **2015**, *71*, 3-8.
6. Dolomanov, O. V.; Bourhis, L. J.; Gildea, R. J.; Howard, J. A. K.; Puschmann, H. *J. Appl. Cryst.* **2009**, *42*, 339-341.
7. G. M. Sheldrick, *Acta Crystallogr. Sect. A*, 2015, **71**, 3-8.
8. Gaussian 09W, Revision D.01; Gaussian, Inc.: Wallingford, CT, 2009.
9. Gaussian 16, Revision B.01; Gaussian, Inc.: Wallingford, CT, 2016.
10. Becke, A. D. *J. Chem. Phys.*, **1993**, *98*, 5648-5652.
11. Perdew, J. P.; Wang, Y. *Phys. Rev. B*, **1992**, *45*, 13244-13249.
12. Grimme, S.; Antony, J.; Ehrlich, S.; Krieg, H. *J. Chem. Phys.*, **2010**, *132*, 154104/1-154104/19.
13. Grimme, S; Ehrlich, S; Goerigk, L. *J. Comput. Chem.*, **2011**, ***32***, 1456-1465.
14. Rappoport, D; and F. Furche, F. *J. Chem. Phys*. **2010**, *133*, 134105/1-134105/11.
